# Formation and Properties of Thermistor Chips Based on Semiconductor 3D Metal Oxide Films Obtained by RF-Magnetron Sputtering

**DOI:** 10.3390/ijms24010742

**Published:** 2023-01-01

**Authors:** Valery Novozhilov, Alexey Belov

**Affiliations:** Research Institute of Physics and Technology, National Research Lobachevsky State University of Nizhny Novgorod, 603022 Nizhny Novgorod, Russia

**Keywords:** thermistor, RF-magnetron sputtering, electrical properties, spinel films

## Abstract

The formation of oxide semiconductor films of the (Mn,Co,Cu)_3_O_4_ type by radio frequency magnetron sputtering is presented. The conditions of deposition and subsequent heat treatment make it possible to obtain films with electrophysical characteristics close to those of the bulk ceramic materials used as a target for magnetron sputtering. Two variants of thermistor geometry were implemented. In the first case, the working layer of oxide semiconductor was deposited directly on the dielectric substrate (planar geometry), and in the second case on the layer with high electrical conductivity (Ni or Al) forming the inner electrode (layered geometry). The lower limit of the nominal resistance of the planar thermistor while maintaining high temperature nonlinearity is ~ 10 kΩ. The layered structure with the inner electrode makes it possible to reduce the lower limit of resistance up to ~ 50 Ω without losing the temperature nonlinearity of the thermistor. In addition, heat treatment above 450 °C or current self-heating with sufficient power output leads to the appearance of a pronounced voltage nonlinearity, which increases the thermal constant *B* of thermistors from 2400–3400 to 5000–5500 K. The fields of application of oxide-film structures for the correction of linear resistors and the implementation of integration approaches in the construction of linearized sensors are discussed.

## 1. Introduction

Multicomponent heterogeneous systems based on transition metal oxides, depending on the methods of their preparation and microstructure, exhibit various electrical instabilities and nonlinear effects. In the applied aspect, structures based on transition metal oxides, which have nonlinear effects of thermal and field conduction, have already found wide application as various electronic devices, primarily in the form of thermistors and varistors. The vast majority of the most common industrial types of thermistors are based on 3D transition metal oxides. They have a wide variety of electrical properties, which makes it possible to develop thermistors with different character of the temperature dependence of resistance [1,2,3,4].

The prevailing approach for obtaining nonlinear semiconductor resistors (NSRs) is based on traditional ceramic technology [3]. At the same time, it is of great practical interest to implement NSRs with a two-dimensional configuration using planar device technology. The use of thin-film technology for creation of NSRs provides a number of advantages compared to traditional ceramic technology—reduced dimensions, weight and thermal time constant, extended range of resistance values, etc. Thin-film thermistors are grown using various methods, such as the chemical solution deposition [5], pulsed laser deposition [6], screen printing [7], electron-beam evaporation [7] and radio-frequency (RF) magnetron sputtering [7,8,9].

The general regularity of the thermistor material characteristics depending on the composition of the oxide semiconductor ceramics is that the highest values of the temperature coefficient of resistance α (TCR) have materials with a high resistivity. With a decrease in resistance, the nonlinear properties drop significantly. Therefore, obtaining thermistors with low resistance (less than 100 Ω) and high temperature sensitivity is not an easy task.

Classical thermistor compositions such as spinel (Ni,Mn)_3_O_4_, (Ni,Mn,Co)_3_O_4_ and (Ni,Mn,Fe,Co)_3_O_4_ cover the largest range of resistivity [10] and are a good basis for the formation of thin-film coatings with a wide variety of electrophysical properties.

The purpose of this work is obtaining semiconductor films of 3D transition metal oxides with electrophysical parameters close to the bulk ceramic material with the RF-magnetron sputtering method and the formation on this basis of thermistor chips characterized by low resistance (tens and hundreds of Ohms) while maintaining high temperature non-linearity. Obtaining relatively low resistance and high temperature non-linearity can be achieved by formation of a layered thermistor structure. Two designs of thin-film thermistor chips were realized. In the first design, the working layer of the oxide semiconductor is deposited directly on the dielectric substrate (planar geometry), and in the second design on a layer with high electrical conductivity (Al or Ni) forming the inner electrode (layered geometry). In thin-film structures based on semiconductor oxides of transition metals, the effects of not only thermal but also field nonlinearity are manifested. These structures are promising for use as various electronic devices and sensitive elements of sensor systems. Different applications of semiconductor oxides in combination with resistive layers of linear resistors are discussed.

## 2. Results and Discussion

### 2.1. Films of Transition Metal Oxides

Thin films (0.5–2 μm) of thermistor material were deposited on polycor (Al_2_O_3_) substrates by RF-magnetron sputtering from (Mn,Co,Cu)_3_O_4_-type ceramic targets with Ni or Cr additives in the mixed Ar (80%) and O_2_ (20%) atmosphere at the substrate temperature of 300 °C or at room temperature. The deposition pressure was 0.5 Pa and deposition rate was 0.5 μm per hour. The targets were disks cut from massive ceramic thermistor materials with three spinel compositions and resistivity *ρ*_v_ from 2.5 to 140 Ω∙cm. After deposition, thermal stabilizing annealing was carried out in air at temperature of 200–800 °C for 2 h.

**Figure 1:**
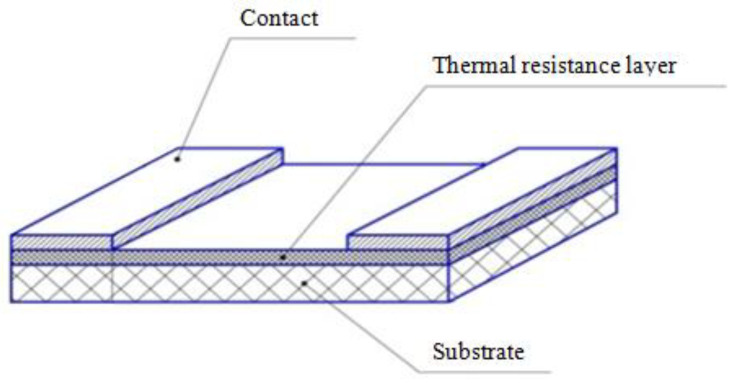
Schematic representation of the thin-film thermistor structure.

Test samples were formed by depositing nickel contacts and cutting the substrate into 3.2 × 1.6 mm elements with 0.5 mm contacts (Figure 1).

Table 1 lists the elemental composition of three ceramic materials of targets, as well as the composition of films with a thickness of 1.5 μm, deposited from the corresponding ceramics, determined by X-ray spectral analysis. It can be seen that the cationic composition of oxide phases in the film material is close to the composition of ceramic target. In other words, the film material inherits the cationic composition of the oxide phases of ceramic target.

**Figure 2:**
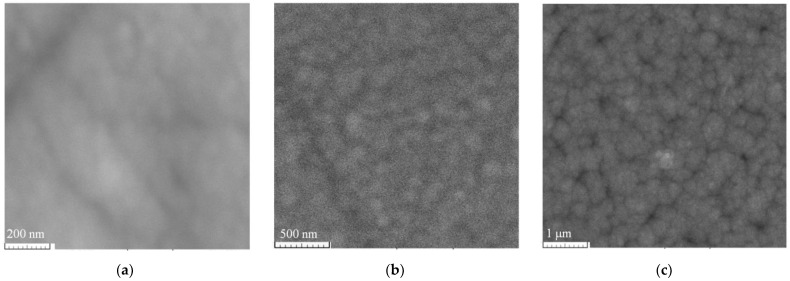
Scanning electron microscope images of films obtained from 6.5 Ω∙cm ceramic at substrate temperature: (**a**) without heating; (**b**) 300 °C; (**c**) 300 °C followed by annealing at 600 °C.

The substrate temperature during deposition has a significant effect on the microstructure of the formed films. The films prepared without heating are amorphous (Figure 2a). The deposition at 300 °C led to the appearance of a polycrystalline structure with an average grain size of 0.2 μm (Figure 2b). After subsequent annealing at a temperature of 600 °C (1 h), the average crystallite size increased to 0.35 µm (Figure 2c).

Films formed without substrate heating have low reproducibility and stability of electrophysical characteristics. The substrate temperature above 300 °C has a decisive effect on the defectiveness and microstructure (Figure 2).

The temperature dependence of the semiconductor material resistance largely determines the properties of the NSR. The electrical conductivity of the film based on 3D transition metal oxides varies exponentially in a wide temperature range. With two known resistance values corresponding to temperatures *T*_1_ and *T*_2_, the expression for the temperature dependence of the resistance has the form:

(1) $$R_{1}=R_{2}\exp B\left(\frac{1}{T_{1}}-\frac{1}{T_{2}}\right),$$

where *R*_1_—resistance at temperature *T*_1_; *R*_2_—resistance at temperature *T*_2_; *B*—coefficient of temperature sensitivity (constant coefficient depending on the properties of the thermistor material) [2]:

(2) $$B\left(K\right)=\frac{1}{\left(\frac{1}{T_{1}}-\frac{1}{T_{2}}\right)}\cdot \ln\left(\frac{R_{1}}{R_{2}}\right).$$

**Figure 3:**
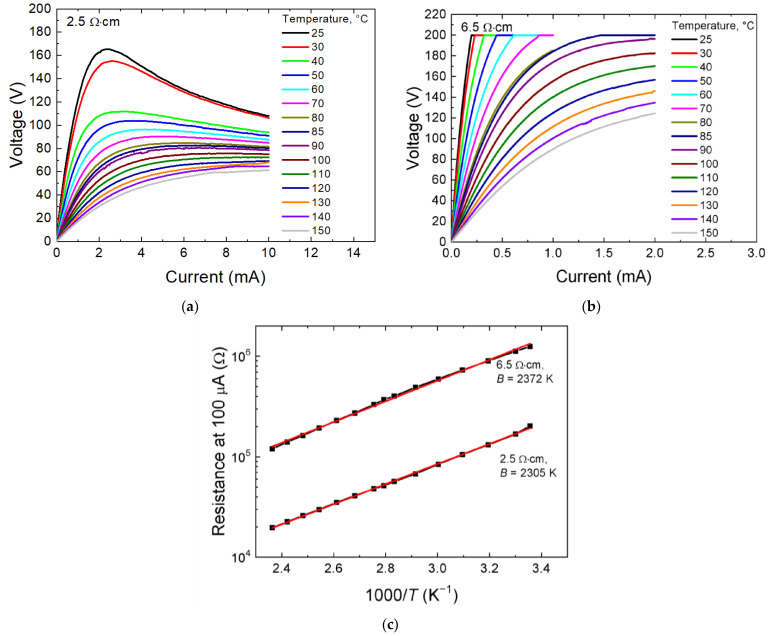
*I*–*V* characteristics at different measurement temperatures of the films obtained from ceramics 2.5 (**a**) and 6.5 Ω∙cm (**b**); (**c**) the dependence of resistance in logarithmic scale on the reciprocal temperature.

Figure 3a,b show the *I*–*V* characteristics in the temperature range 25–150 °C of the films prepared from 2.5 and 6.5 Ω∙cm ceramics at substrate temperature 300 °C. The coefficient of temperature sensitivity *B* found from the Ln(*R*)–1/*T* slope (Figure 3c) is 2305 and 2372 K, respectively, and the activation energy *E*_a_ = *B*∙*k*, where *k,* the Boltzmann constant, is ~ 0.20 eV.

In contrast to bulk ceramic materials, which have a complex dependence of conductivity on the annealing temperature [1], the studied film structures exhibit only an increase in conductivity with an increase in the annealing temperature up to 800 °C. Heat treatment in the atmosphere at 500–600 °C for 40–60 min is sufficient to stabilize the structural-phase state of the film material close to that of a bulk ceramic material.

The properties of the obtained films correlate well with the calculated estimates based on the properties of the bulk material. Estimates of the nominal resistance of film structures with material resistivity corresponding to the used ceramic targets and experimental values are given in Table 2.

Thus, the RF-magnetron sputtering method makes it possible to form semiconductor films with pronounced thermistor properties, which manifest themselves both immediately after sputtering and after heat treatment. When changing the target with a lower or higher resistivity and use film thickness range of 0.3 to 2 μm, it is possible to obtain resistance values from tens of kΩ to hundreds of MΩ while maintaining the property of temperature nonlinearity at the level of bulk ceramic samples, which makes it possible to create working coatings for passive electronic components that meet a variety of functional requirements. For most practical applications of thermistor layers, deposition can be limited to a single target with a resistivity of about 100 Ω∙cm.

### 2.2. Thermistors

Oxide compounds with a spinel structure have increased resistance to thermal and electrical loads and provide a wide range of resistivity values at negative TCR α_25_ of 1% K^−1^ and higher. In addition, the low sensitivity of electrical conductivity to large changes in the cationic composition of the material makes the formation of thin films from these materials more technological and more reproducible.

The achievable range of nominal resistance in the classical planar configuration, while maintaining high nonlinearity, begins with values of 8–10 kΩ and higher. However, the formation of thin-film NSRs in a chip version implies their wider use in low-voltage functional blocks of equipment, which requires a decrease in thermistor resistance down to tens and units of ohms with a decrease in operating voltages. The combination of relatively low resistance and high nonlinearity requires a significant reduction in thickness and increase in the cross section of the working volume of the thermistor, which can be achieved by transition to a layered thermistor design.

Two designs of thermistor chip were implemented. In the first case, the working layer of oxide semiconductor was deposited directly on the dielectric substrate (planar geometry), and in the second case on the film with high electrical conductivity forming the inner electrode (layered geometry).

The first design of the thermistor chip included the following technological operations:

- RF-magnetron sputtering semiconductor film with a thickness of 0.3–2 µm in the atmosphere Ar + O_2_;
- Annealing at 500–600 °C to stabilize the structural-phase state of the working material;
- Deposition of nickel contacts with the thickness of ~ 0.3 µm and *ρ*_s_ < 1 Ω/□ by electron-beam evaporation.

**Figure 4:**
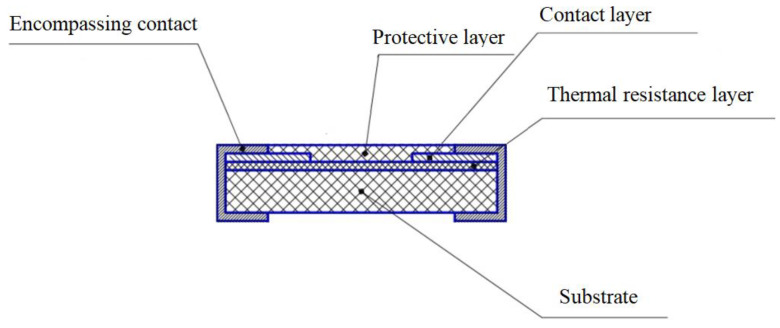
Schematic representation of the thermistor chip in planar geometry.

A schematic representation of the planar geometry of thermistor chip is shown in Figure 4.

**Figure 5:**
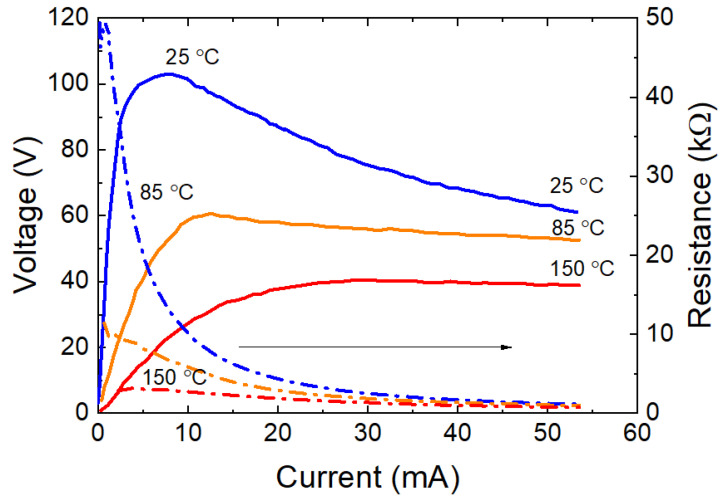
Temperature dependence of *I*–*V* characteristics of thermistor chip in planar geometry obtained from ceramic target 140 Ω∙cm. *R*_25_ = 47 kΩ, *α*_25_ = −3.84% K^−1^.

The planar thermistor design fully complies with linear resistor manufacturing technology and provides a nominal thermistor resistance from units of kΩ to hundreds of MΩ using a relatively low resistivity ceramic target (less than 150 Ω·cm). Figure 5 shows typical current–voltage characteristics of planar thermistors obtained from a 140 Ω∙cm ceramics. The resistance at 25 °C is about 47 kΩ, and TCR is −3.84% K^−1^.

Within this geometry, as studies of thermistor films show, it is difficult to achieve nominal resistance values of tens and hundreds of ohms.

The second design of the thermistor chip is a metal–semiconductor–metal layered structure, when the geometry of the current path is determined by the film thickness and the area of the contact pads. This approach seems to be promising for achieving low nominal resistance while maintaining high nonlinear properties.

An inner conductive layer is introduced into the design of the thermistor, which is completely covered with the semiconductor material film. This thermistor design introduces fundamentally new moments in the characteristics of the thermistor.

The second design of the thermistor chip included the following technological operations:

- Deposition on a dielectric substrate of metal film (Ni or Al) with a thickness of ~ 0.2 μm and surface resistance of a few ohms;
- Formation of the desired configuration of the inner conductive layer by photolithography or the free mask method;
- RF-magnetron sputtering of semiconductor film with a thickness of 1–2 μm;
- Annealing at 300–400 °C to stabilize the structural-phase state of the working material;
- Deposition of nickel contacts by electron-beam evaporation through a photolithographic mask (explosive photolithography method). The contact thickness is ~ 0.3 µm, resistance is *ρ*_s_ < 1 Ω/□, and the size variation across overlap with bottom electrode is from 0.5 to 1.5 mm.

**Figure 6:**
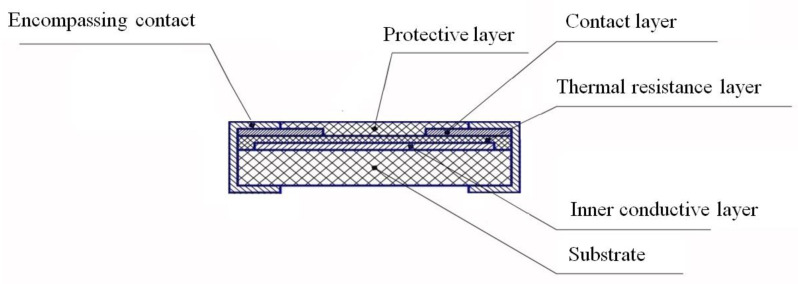
Schematic representation of the thermistor chip in layered geometry.

A schematic representation of the layered geometry of thermistor chip is shown in Figure 6.

**Figure 7:**
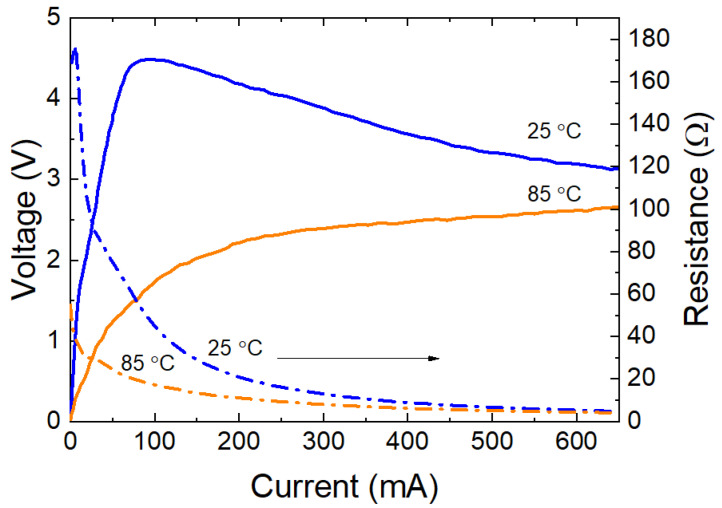
Temperature dependence of *I*-*V* characteristics of the thermistor chip in a layered geometry with Ni inner layer obtained from the ceramic target 140 Ω∙cm. *R*_25_ = 178 Ω, α_25_ = −3.64% K^−1^.

The layered structure of the thermistor at operating voltages less than 10 V (in most cases less than 5 V) is characterized by currents up to 500–1000 mA (Figure 7). The minimum value of nominal resistance for thermistors with high temperature non-linearity was 25–50 Ω. The main factor preventing the reduction in the thermistor resistance to a few ohms is the transition resistance at the interface of the semiconductor with metal electrodes.

For a metal–semiconductor contact, a nonlinear behavior usually takes place, and its electrical conductivity is determined by many processes—thermionic emission, tunneling, impact ionization, thermal ionization of impurities and defects, etc. As a result, the metal–semiconductor contact has a metal-transition layer-semiconductor structure.

During the annealing of a semiconductor film, significant changes occur in the parameters of the transition layer. As a result, the behavior of the transition barrier layer and its characteristics become important for realizing the desired performance of the thermistor as a whole. Non-ohmic properties appear, which are expressed by a strong dependence of the electrical conductivity on the field strength (varistor effect), which depends both on the inner electrode material and on technological factors, primarily on the annealing temperature and time. The value of the annealing temperature near 450 °C becomes critical in changing the behavior of the layered structure.

Thus, for a layered thermistor chip with the Al inner electrode, a pronounced field dependence of the potential barrier height began to appear at film annealing temperatures of 420 °C and higher. Thus, the resistance of thermistors obtained from the ceramic target of 6.5 Ω∙cm varies from 923 to 5.8 kΩ in the voltage range of 0 to 3 V. The temperature dependence of the resistance, taken at voltages of 0.1 and 3 V (when the thermal mechanism of resistance drop is not yet working), shows the values of the constant of temperature sensitivity *B*_25–85_ = 4706 and *B*_25–85_ = 2886 K, and the TCR is −5.3% and −3.25% K^−1^, respectively.

For the thermistor with the Ni inner electrode, the field dependence of the potential barrier height began to manifest itself at a higher annealing temperature of 500 °C. The coefficients of temperature sensitivity at voltages of 0.1 and 2 V are *B*_25–85_ = 4170 and *B*_25–85_ = 2310 K, and the TCR is −4.7% and −2.6% K^−1^ respectively. These results point to the importance of finding controllable combinations of semiconductor oxide material and metal electrodes in order to provide reproducible parameters of thermistor structures.

With an increase in the electric field, the resistance of the thermistor layer depends less on measurement temperature, which is explained by the field dependence of the potential barrier height at the metal–semiconductor interface. The potential barrier height can be estimated from the relative change in conductivity *φ*_0_ = Δ Ln *σ*(*T*)·*kT* = 0.14 eV. This value is close to the activation energy of the electrical conduction process in semiconductor oxides of transition metals, which form the basis of thermistor materials [1].

**Figure 8:**
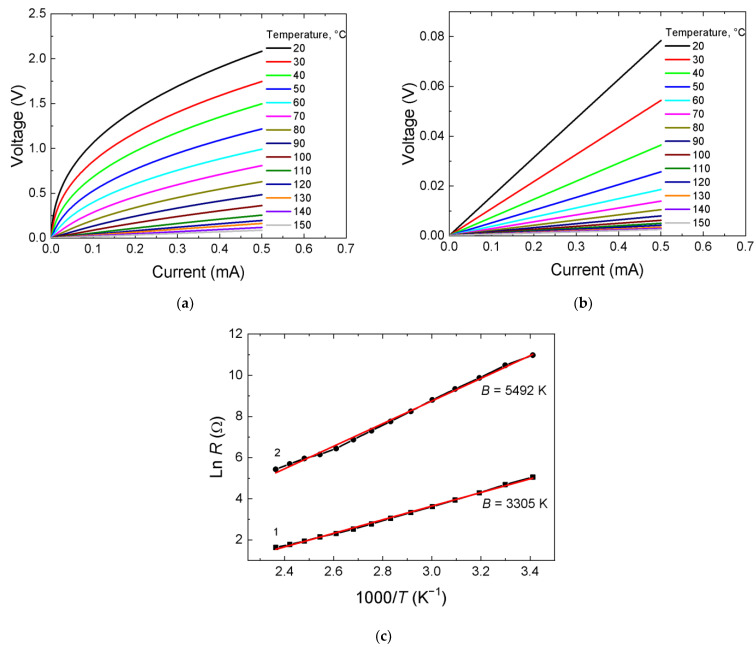
Temperature dependence of *I*–*V* characteristics of the thermistor chips in a layered geometry with Ni inner layer obtained from the ceramic target 140 Ω∙cm and film annealing at 600 (**a**) and 300 °C (**b**); (**c**) Plots of Ln(*R*) vs. 1000/*T* curves of thermistor chips with film annealed at 300 (1) and 600 °C (2).

**Figure 9:**
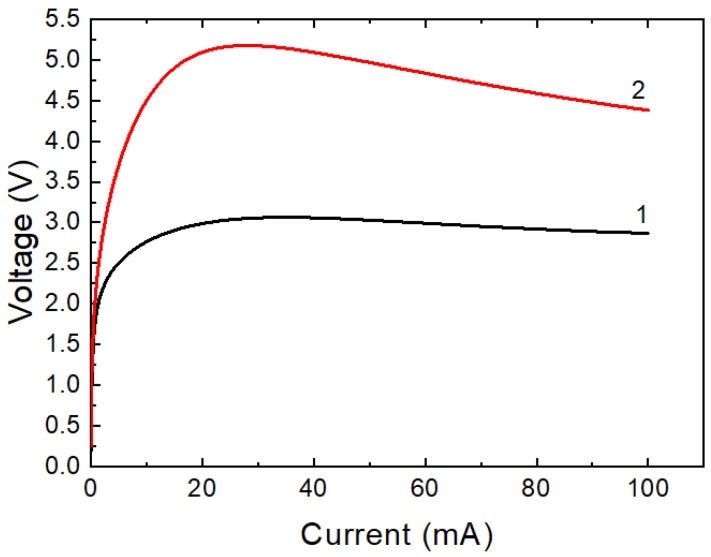
*I*–*V* characteristics at room temperature of layered thermistor chips with Ni inner layer obtained from ceramics of 6.5 (1) and 140 Ω∙cm (2) at film annealing at 600 °C.

There is no linear section between the current and voltage at the beginning of *I*–*V* characteristics of layered structures annealed at temperatures of more than 400 °C (Figure 8a), since the strong dependence of the electrical conductivity on the field strength is manifested, in contrast to structures annealed at lower temperatures (Figure 8b). The coefficient of temperature sensitivity increases from 3000–4000 to 5000–5500 K (Figure 8c). The *I–V* characteristic of the thermistor takes on a “varistor” form with the stabilization voltage of 3–5 V (Figure 9). Even high-resistance samples with a static resistance of hundreds of kΩ show avalanche-like self-heating with a maximum voltage of less than 8 V.

A similar transformation of the electrical properties of the layered thermistor can be achieved at operating currents that ensure heating of the samples to significant temperatures. An electrical load of 3–5 W for about 10 s gradually increases the nominal resistance of the thermistor. By repeating the loading procedure many times, the resistance changes from the initial tens of Ohms to tens of kOhms. After current heating, as with after annealing, voltage nonlinearity appears, which increases the coefficient of temperature sensitivity from 3000–4000 to 5000–5500 K.

Thus, the metal-oxide semiconductor–metal heterostructure allows:

- Low resistance values while maintaining the high temperature non-linearity of the thermistor;
- High load capacity of thermistors, an average of 4–6 W at a voltage not exceeding 10 V;
- Increased effective value of the coefficient of temperature sensitivity *B* to values of 5500 K;
- Expansion of the possibilities and applicability of integration approaches in the development of electronic components.

### 2.3. Thin-Film Resistors

Consider the effect of an additional thermistor layer on the characteristics of thin-film linear resistors.

The main factor in the degradation of the film resistor during operation is local thermal overheating associated with various inhomogeneities of the resistive layer. The exclusion or reduction in the destructive effect of local thermal inhomogeneities of the resistive layer can be achieved by using an additional layer of thermistor material with the required parameters. The most important property of such a coating is that it practically does not manifest itself under normal operating conditions of the resistor due to the high nominal resistance in the operating temperature range. When critical overheating of the working layer occurs, the conductivity of the thermistor material increases, which leads to an effective mechanism of current redistribution and subsequent equalization and suppression of thermal inhomogeneity. The load capacity and therefore the reliability of the resistor increase many times.

To estimate the parameters of a layered system of resistive and additional thermistor semiconductor films, we used the equations for the surface resistivity *R* of a binary system depending on the surface resistivity *R*_1_ and *R*_2_ and the TCR *α*_1_ and *α*_2_ for the resistive and additional layers, respectively:

(3) $$R=\frac{R_{1}\cdot R_{2}}{R_{1}+R_{2}},$$

(4) $$\alpha =\frac{R}{R_{1}}\alpha_{1}+\frac{R}{R_{2}}\alpha_{2}.$$

Index 1 characterizes the surface resistivity and TCR of the resistive layer, and index 2 refers to the corresponding parameters of the additional layer.

The mechanism of effective current redistribution in the region of local thermal overheating is realized if the conductivity of the additional layer is higher than the conductivity of the resistive layer at a temperature that causes accelerated aging processes. For most resistive materials, temperatures in the range of 350 to 500 °C are thresholds for activation of various diffusion processes, defect annealing, and structure relaxation.

Assume that the temperature of local overheating (*T*_2_), which requires protection activation, is 400 °C, and the coefficient of temperature sensitivity of the additional layer material *B* = 2400 K, then from the ratio

(5) $$R_{T}=R_{2}exp\left(\frac{B\cdot \left(T_{1\;}-T_{2}\right)}{T_{1\;}\cdot \;T_{2}}\right),$$

where *T*_1_ = 25 °C is the initial temperature value, *R*_2_ is the surface resistivity of the additional layer at *T*_1_ and *R_T_* is the surface resistivity of the additional layer at *T*_2_, which is equal to the surface resistivity of the resistive layer (*R_T_* = *R*_1_), and we have *R*_2_/*R_T_* = 89, and at *R*_1_ = 220 Ω/□ we obtain *R*_2_ = 19.6 kΩ/□.

Similar estimates are presented in Table 3 for typical characteristics of the additional thermistor layer based on overheating temperatures of 400 and 500 °C and NiCr film with *α* = +2 × 10^−4^ K^−1^.

The load capacity of resistors based on NiCr resistive material with surface resistivity from 20 to 380 Ω/□ was tested on resistor samples with dimensions of 3.2 × 1.6 mm in the resistance range from 24 to 12 kΩ. The tests were carried out using direct current with increasing loads and holding for 1 min at each step of 0.2 W, removing the load, measuring the nominal resistance, etc., until the sample failed.

The power dissipation range for standard resistors, leading to a complete failure of the sample, was from 0.6 to 1.8 W. Parametric failures were manifested at a load level of 1.2 W. Resistors with an additional thermistor layer did not have parametric failures up to loads of 4.2 W, and a complete failure occurred at a power dissipation above 6.6–7.5 W.

The expansion of the allowable operational impacts for resistors with an additional layer is quite clearly manifested in the behavior of the samples under the influence of increasing loads. Providing a larger margin for thermal dissipation directly affects the increase in the reliability of the resistor.

Assume that the resistive film has a positive TCR of a known value, then from Equation (5), by the condition of minimizing the TCR of a two-layer system, the following relation is obtained:

*R*_2_/*R*_1_ = *α*_2_/*α*_1_, (6)

which can be used to estimate the values of the surface resistivity *R*_2_ of the additional layer from the known value of the surface resistivity and TCR of the resistive film (see Table 3).

Let us consider the temperature behavior of the resistive film with the semiconductor layer from the condition of minimizing the resistance change in the two-layer system within a given temperature range:

(7) $$\Delta R=\frac{(R_{1}+\Delta R_{1\;})\cdot R_{2}\left(T\right)}{R_{1\;}+\Delta R_{1\;}+R_{2}\left(T\right)}-\;\frac{R_{1\;}\cdot R_{2}}{\left(R_{1\;}+R_{2}\right)}$$

where *R*_1_ and *R*_2_ are the surface resistivity of the resistive and additional layers at the initial temperature *T*_1_, for example, 25 °C; *R*_2_(*T*) is the surface resistivity of the additional layer, changing in accordance with (5) at *T*_2_, for example, 100 °C; and Δ*R*_1_ = *α*_1_·Δ*T*–change in the surface resistivity of the resistive film in the temperature range Δ*T* = *T*_2_ − *T*_1_ = 75 °C.

Taking into account that Δ*R*_1_ << *R*_1_, *R*_1_ + Δ*R*_1_ + *R*_2_(*T*) ≈ *R*_1_ + *R*_2_(*T*) and according to the condition that expression (7) is equal to zero after transformations, we obtain the following equation:

(8) $$\frac{R_{2}}{R_{1}}=\frac{R_{2}-R_{2}\left(T\right)}{R_{2}\left(T\right)}\cdot \frac{R_{1}}{\Delta R_{1\;}},$$

or considering that $\frac{\Delta R_{1\;}}{R_{1}}=\alpha_{1}\cdot \Delta T$ expression (8) takes the form:

(9) $$\frac{R_{2}}{R_{1}}\;=\left[exp\left(\frac{B\cdot \Delta T}{T_{1\;}\cdot T_{2}\;}\;\right)-1\right]/\alpha_{1}\cdot \Delta T,$$

Expression (9) contains the temperature interval Δ*T* as a parameter within which there is maximum Δ*R* of the two-layer system, while the TCR still remains positive. This estimate can be useful in thermal compensation problems.

Relation (6) determines the condition for achieving TCR equal to zero, which means a change in the sign of TCR from positive to negative. Relation (6) is a more stringent criterion compared to (9).

**Figure 10:**
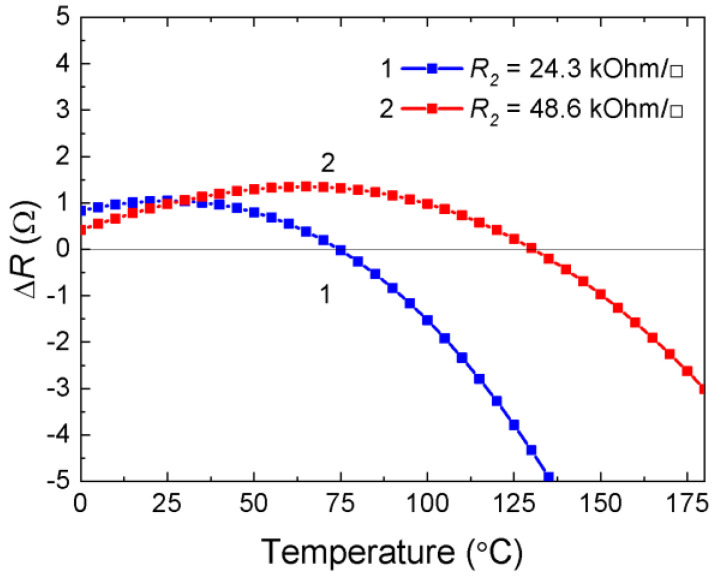
Calculated dependence of the temperature change in the resistance of a two-layer system according to criteria (6) and (9).

Based on the known *R*_1_ and *α*_1_ of the resistive film, the value of the surface resistivity *R*_2_ of an additional layer is estimated. An illustration of the behavior of a two-layer structure is shown in Figure 10. Given the parameters of NiCr resistive film: *R*_1_ = 180 Ω/□, α = +2.1 × 10^−4^ K^−1^; semiconductor layer: *B* = 2400 K, we obtain *R*_2_/*R*_1_ = 135 and *R*_2_ = 24.3 kΩ/□ according to criterion (6) and *R*_2_/*R*_1_ = 270 and *R*_2_ = 48.6 kΩ/□ according to criterion (9). This determines the temperature behavior shown in Figure 10 curves (1) and (2), respectively.

The large negative TCR of the thermistor material of the additional semiconductor layer makes it possible to compensate any positive TCR of the resistive layer. The TCR of layered system changes in a given temperature range from a positive value inherent in the TCR of a resistive film, passes through zero at the boundary of the temperature range, and then inevitably takes increasing negative values depending on the parameters of the additional semiconductor layer.

The proposed approach for modifying linear resistors can be extended to the development of integrated temperature sensors and thermal compensation elements, creating all the necessary passive components for serial and parallel connection in a single process.

## 3. Methods

The composition of the deposited films was determined by X-ray spectral analysis on a Tescan Vega II scanning electron microscope with an Energy 250 spectrometer attachment. The surface microstructure of the films was characterized using the same equipment.

The study of electrophysical characteristics was carried out using an Agilent B1500A semiconductor device analyzer. The samples were placed in a hermetically sealed metal thermostat. The measurements were carried out in air in the temperature range 20–150 °C using the isothermal measurement mode. The temperature was maintained with an accuracy of 1 °C.

## 4. Conclusions

The RF-magnetron sputtering method makes it possible to form films of semiconductor oxide phases with composition, resistance and coefficient of temperature sensitivity characteristics close to those of the ceramic target material. The layered geometry of thermistor with the inner electrode makes it possible to reduce the lower limit of resistance up to ~ 50 Ω without losing the temperature nonlinearity. Heat treatment above 450 °C or current self-heating with sufficient power output leads to the appearance of a pronounced voltage nonlinearity, which increases the thermal constant *B* of thermistors from 2400–3400 to 5000–5500 K. The use of an additional thermistor layer in the design of linear resistors can increase the load level at which the failure occurs.

## Figures and Tables

**Table 1:** Elemental composition of ceramic materials used for the manufacture of targets, as well as the composition of deposited films with a thickness of 1.5 μm.

	Weight Fraction of the Element, %				
	Mn	Co	Cu	Ni	Cr
ceramics 2.5 Ω∙cm	44.5	9.1	31.17	-	3.2
film	48.8	12.5	28.9	-	3.0
ceramics 6.5 Ω∙cm	31.2	9.7	17.0	-	-
film	34.6	11.2	17.7	-	-
ceramics 140 Ω∙cm	26.8	35.3	4.4	2.8	-
film	31.1	36.5	5.9	2.2	-

**Table 2:** Surface resistivity and nominal resistance of thermistor films, calculated for a thickness of 1 µm, as well as experimental values of the thermistor film resistance.

Resistivity of Ceramics *ρ_v_*_,_ Ω∙cm	Temperature Sensitivity Coefficient *B*, K	Surface Resistivity of the 1 µm Thick Ceramic Layer, *R_s_*, kΩ/□	Estimated Nominal Resistance of Thermistor Films, kΩ	Experimental Value of the Thermistor Films Resistance with a Thickness of 1–2 μm, kΩ
2.5	2020	25	36.3	8–255 *
6.5	2400	65	94.5	30–1200 *
140	3400	1400	2036	660–12,400 *

* Upper limit of resistance corresponds to samples without annealing.

**Table 3:** Estimation of the resistance ratio of the resistive and additional semiconductor layers based on overheating temperatures of 400 and 500 °C.

*B*, K	*R*_2_/*R*_1_ at Overheating Temperature 400 °C	*R*_2_*/R*_1_ at Overheating Temperature 500 °C	*R*_2_*/R*_1_ According to the Criterion of TCR Minimization
2020	43	65	115 (226 *)
2400	89	141	135 (270 *)
3400	578	1107	190 (591 *)

* *R*_2_/*R*_1_ values according to criterion (9).

## Data Availability

Not applicable.
